# Beyond Laparotomy: Multimodal Strategies and Emerging Evidence in Minimally Invasive Management of Gastric Gastrointestinal Stromal Tumors

**DOI:** 10.1155/bmri/6305081

**Published:** 2026-03-08

**Authors:** Wenjing Sun, Song Zhao

**Affiliations:** ^1^ Department of Gastroenterology, The Thirteenth People′s Hospital of Chongqing (Chongqing Geriatrics Hospital), Chongqing, China; ^2^ Department of General Surgery, The Thirteenth People′s Hospital of Chongqing (Chongqing Geriatrics Hospital), Chongqing, China

## Abstract

**Background:**

Gastrointestinal stromal tumor (GIST) is a type of potentially malignant mesenchymal tumor, thought to arise from the interstitial cells of the gut. More than half of all GIST cases occur primarily in the stomach. Currently, radical resection remains the only curative treatment for this disease. This review is aimed at summarizing recent advances in surgical management and operative techniques for resectable gastric GISTs.

**Summary:**

Risk stratification of small gastric GISTs is expected to resolve ongoing debates regarding their management, while artificial intelligence (AI)–assisted imaging shows promise for early detection. In tumors located at unfavorable sites, R1 resection has been shown to achieve oncological outcomes comparable to those of R0 resection, thereby expanding surgical options. The principles of en bloc resection and avoidance of tumor rupture remain critical in intraperitoneal surgery. However, their applicability in endoscopic resection (ER) remains uncertain. Clinical studies have confirmed the long‐term oncological safety and superior short‐term outcomes of laparoscopic resection (LR), even for tumors larger than 5 cm. For LR in challenging locations, expert centers, robotic assistance, and laparoscopic intragastric surgery represent viable alternatives. Innovations and integration of endoscopic techniques have improved the efficiency and feasibility of ER for gastric GISTs, while also reducing complication rates. As an emerging approach, laparoscopic endoscopic cooperative surgery (LECS) combines the advantages of both LR and ER, offering reliable oncological control with a lower incidence of complications.

**Summary**

• The emergence of new clinical evidence has significantly advanced the surgical management of gastric stromal tumors.
• However, ongoing challenges necessitate further in‐depth investigation.

## 1. Introduction

Gastrointestinal stromal tumor (GIST) is a type of mesenchymal tumor with malignant potential, believed to originate from the gut′s interstitial cells. Most GISTs express biomarkers such as ANO1 and KIT [1, 2], which are also specifically detected in the interstitial cells of Cajal (ICCs). Additionally, platelet‐derived growth factor receptor alpha (PDGFRA) had been identified in a subgroup of GISTs [3]. It is reported that the smooth muscle cells (SMCs), ICCs, and PDGFRA‐positive cells form the SIP syncytium, a structure essential for gut motility [4, 5]. However, definitive evidence linking GISTs to motor dysfunction in the gut remains elusive. The widespread adoption of computed tomography (CT) and gastric endoscopy over the past two decades has led to a significant increase in GIST detection.

More than half of all GISTs are reported to occur in the stomach [2, 6]. Radical resection remains the only curative treatment for GISTs. This review will focus on recent advances in the surgical management of resectable gastric GISTs and discuss the latest operative techniques in this field.

## 2. Advances in Surgical Management

### 2.1. Small Gastric GIST

For gastric GISTs larger than 2 cm or those that have led to acute abdomen presentations such as perforation, bleeding, or obstruction, resection with or without targeted drug therapy is generally recommended [7]. Nevertheless, no consensus is made in terms of the optimal treatment for small gastric GISTs (tumor size < 2 cm). Given their typically indolent behavior, a strategy of close follow‐up surveillance is usually advised [8, 9]. However, several high‐risk factors, including ulcer formation, irregular borders, invasion of adjacent structures, and heterogeneity, should be excluded before finalizing management [10].

In contrast, some guidelines also propose surgical resection as an option for selected small gastric GISTs [11, 12]. A recent retrospective study revealed a 5‐year GIST‐specific mortality rate of 12.9% among patients with small GISTs and no concurrent malignancies [13].

In any case, the fundamental clinical challenge lies in accurately assessing the malignant potential of small gastric GISTs preoperatively, thereby avoiding both overtreatment and delayed intervention. Artificial intelligence (AI)–assisted imaging examinations may be very promising tools in risk stratification [14, 15].

### 2.2. R1 Resection

The complete resection (R0) is always the optimal surgical treatment for any resectable gastric GISTs. However, in some cases, R1 resection, defined as the presence of positive microscopic margins, may be detected pathologically. Patients undergoing R1 resection were recommended for adjuvant treatment with tyrosine kinase inhibitors (TKIs), such as imatinib [16]. This approach was based on the premise that TKIs could reduce the risk of recurrence by targeting residual microscopic disease.

Increasing evidence has documented that the R1 resection without adjuvant treatment (e.g., imatinib) may be an alternative suboptimal choice for gastric GISTs [17–24]. These results may provide more surgical modality options for the surgical management of gastric GISTs located in unfavorable sites [25] such as the esophagogastric junction (EGJ), pyloric ring, posterior wall, antrum, and lesser curvature. As surgeons have always followed the rule that it is imperative to prioritize functional preservation, complex surgeries such as the total or subtotal gastrectomy with or without multivisceral resection should be cautiously avoided unless necessary. Nevertheless, it is crucial to emphasize the importance of avoiding intraoperative tumor rupture (discussed later), as it carries a more unfavorable prognosis than the R1 resection.

Although R1 resection is currently acceptable, this does not mean that surgeons should abandon the pursuit of R0 resection. This principle mainly supports that when postoperative pathology confirms an R1 resection, additional surgery is not mandatory, especially for cases where further surgery could result in severe organ damage, significantly increase postoperative complications, or impose a heavy economic burden. Meanwhile, the patient′s wishes should also be taken into account under fully informed circumstances.

### 2.3. Tumor Rupture

The newly defined tumor rupture includes tumor fracture or spillage, blood‐stained ascites, gastrointestinal perforation at the tumor site, microscopic infiltration of an adjacent organ, intralesional dissection or piecemeal resection, and incisional biopsy [26]. It has been reported that the intraoperative tumor rupture is a high‐risk factor for relapse [20, 27]. Therefore, the prevention of tumor rupture is a key consideration in surgical strategy, particularly to achieve en bloc removal during intraperitoneal surgery. To achieve en bloc removal, iatrogenic tumor rupture must be avoided at all costs. For example, maintain the integrity of the pseudocapsule throughout the entire procedure, remove the tumor along with some normal tissue, avoid piercing the tumor with surgical instruments, avoid laparoscopic surgery if the tumor integrity cannot be kept, and immediately place the tumor into a specimen bag for isolation once removed.

To avoid tumor rupture during intraperitoneal surgery, endoscopic resection (ER) is a feasible alternative surgical approach. A tumor size‐related indication for ER is advocated, given the unfeasibility of en bloc removal of the larger specimen endoscopically [28]. To ensure the integrity of specimens, tumor sizes smaller than 3 cm are put in collection bags and retrieved orally, while tumor sizes larger than 3 cm are retrieved via the laparoscopically [29]. Recently, evidence has shown that ER for gastric GISTs with tumor size ≥ 5 cm is technically feasible. It not only achieves a short‐term prognosis similar to laparoscopic resection (LR) but also has the advantages of rapid postoperative recovery and low cost [30]. However, no consensus is made on how to retrieve specimens. Zhang et al. reported a comparable oncological outcome within a mean follow‐up of 4 years in the piecemeal removal and en bloc removal groups [31]. They attributed these insignificant differences to the R0 resection and the acidic environment within the gastric lumen, which might not be suitable for GIST cells. On the other hand, despite piecemeal resection with unimpaired oncological outcomes being also reported in some other studies [32–34], various limitations of these studies lead to the conception of piecemeal resection still being challenging and debated. Further studies are needed to validate the safety of piecemeal removal or resection when performing the ER for gastric GISTs. It would be interesting to investigate the biological behavior of GIST cells on the gastric mucosa.

### 2.4. LR

In 1992, the first LR for gastric GIST was reported by Lukaszczyk et al. [35]. Laparoscopic surgery, the representative of minimally invasive surgery, had garnered considerable interest among surgeons for its potential in treating GISTs from then on. Similar to the history of laparoscopic surgery for gastrointestinal cancer, the application of laparoscopy for GISTs was doubted in the early days. The main concerns were the long‐term oncological safety and technical feasibility of radical resection for GISTs with large tumor sizes. With the accumulation of extensive practice, the LR of GIST was proven to be equal to the laparotomy in terms of long‐term oncological outcomes, while superior in short‐term outcomes such as less operation time and bleeding, earlier recovery, shorter hospital stay, and lower rate of complications [36–38].

Given the fragility of GISTs, LR was typically recommended for tumors less than 5 cm. Nonetheless, the pursuit of skilled surgeons to challenge these established dogmas never stopped. Several meta‐analyses revealed that LR for GIST larger than 5 cm is a safe alternative to laparotomy with comparable long‐term outcomes [39–41].

It is full of challenges when performing LR for GISTs located in unfavorable sites. Expert centers and robotic surgery seem to be some of the potential advantages for settling these challenges [25, 42–50]. The laparoscopic intragastric surgery (LIS), which was first reported by Ohashi in 1994–1995 [51, 52], is another alternative for GISTs located in unfavorable sites [53–55]. Briefly, three trocars are inserted through the abdominal wall and anterior gastric wall under the monitor of the gastroscope, and then the LR of the lesion is conducted in the gastric lumen. A modified LIS was reported in 2013–2014 by Dong et al.; with the assistance of endoscopists, only two trocars were needed [56, 57]. When the concept of single‐incision laparoscopic surgery was introduced into the LIS, intragastric single‐port surgery was reported in recent years [58–61].

### 2.5. ER

Guidelines recommend that a small gastric GIST is an indication of ER. Once the tumor volume is enlarged in a short time, or the patient has a strong desire for treatment, ER could be considered [62]. Larger gastric GISTs (2–5 cm) that have not invaded other organs are also considered candidates for ER [11]. The pure ER procedures include traditional endoscopic submucosal dissection (ESD), submucosal tunnel endoscopic resection (STER), endoscopic full‐thickness resection (EFTR), and endoscopic mucosa‐sparing‐lateral dissection (EMSLD).

A hybrid resection technique combining EFTR and ESD was reported [63]. It has been confirmed as a safe and efficient technique for GISTs. In general, a full‐layer resection of the gastric wall with extensive suturing is often necessary. It has been reported that considerable time is required for the suturing, and when the closure of the gastric wall is unachievable, laparoscopic closure should be considered [64]. This is technically challenging for the endoscopists. The currently available methods for closing defects endoscopically include metallic clipping with or without endoloop placement, closure using over‐the‐scope metal clips, and suturing. However, these closure methods may not be successful and effective for substantial defects, particularly when operating in the retroflexed position, where access is inherently challenging. Sachdev et al. described an omental patching via endoscopic techniques to close large and hard‐to‐access defects after EFTR of gastric GISTs [65]. In their study, the omental fat was pulled into the gastric lumen and clipped to the edges of the defect. Continuous endosutures were placed at four different areas in a purse‐string fashion in the surrounding mucosa and cinched leading to successful closure. However, the technique requires the endoscopist to be experienced and proficient in endoscopic closure devices.

Recently, Yamashina et al. reported a case series of gastric GISTs that could be completely resected safely and easily without perforation using ESD in combination with a detachable snare [66]. This so‐called endoscopic inversion and strangulation of the muscle layer and resection (EISMR) consists of endoscopically inverting the muscle layer into the gastric lumen and strangulating the muscle layer with a detachable snare and then followed by resection. In this report, five patients with gastric GISTs underwent EISMR. The median tumor size was 2 cm (range, 1.5–5 cm), and the median procedure time was 93 min (range, 58–120). En bloc resection was achieved in all five cases (100%). Strangulation of the muscle layer with a detachable snare was successful in all five cases, and no perforation was observed. No postoperative gastrointestinal bleeding or any other serious complications occurred in this study. This demonstrated the safety and efficacy of EISMR.

In addition, some experts discussed the feasibility of the preserved mucosa unroofing closure technique after EFTR. In this procedure, a four‐fifth circumferential mucosal incision was made around the lesion after submucosal injection. Submucosal dissection was performed to unroof the overlying mucosa, which was preserved via the remaining one‐fifth circumferential mucosal edge. Then, a mucosal flap was created and turned aside to expose the mass [67]. This technique ensures that the reliability of the closure was enhanced since the tension of the wound had been substantially decreased.

Moreover, advances in endoscopic devices may help to shorten the operation time and improve the safety of ER. A novel full‐thickness resection device (FTRD) contains a tissue helix that can grasp the lesion after exposing the tumor by resecting the overlying mucosa and a premounted electrosurgical snare which can be used in the en bloc resection. The helix was used to expose and capture the tumor. While using forward pressure, the tissue helix knob was rotated clockwise so that the helix would penetrate and retract the tumor into the application cap [68].

Recent advances in ER procedure make it more effective and safe for treating gastric GISTs [69]. In practice, different ER procedures can be combined to ensure safety and reduce operation time. Exposing the lesion, avoiding excessive coagulation, and closing the wound in a relatively short time are the keys to ensuring the integrity of the tumor and decreasing the incidence of ER‐related complications.

### 2.6. Laparoscopic Endoscopic Cooperative Surgery (LECS)

As discussed previously, the cooperation between surgeons and endoscopists may be traced back to the conducting of LIS in the 1990s when they were facing a gastric GIST located in an unfavorable site. The endoscopic team usually played the role of assistant by locating, monitoring, and harvesting the lesion endoscopically. With the combination of ESD, a novel technique, which was called LECS, was then described in 2008 [70]. Since then, various modified LECS procedures have been reported. The details of these procedures are extensively reviewed by others [71–73]. Anyhow, the optimal LECS is not identified yet; the choice of these LECS procedures depends on the availability of experienced endoscopists and surgeons, the characteristics of lesions, and the patient′s overall health status.

A meta‐analysis reviewing 18 single‐arm studies with a total of 702 cases in LECS for gastric GISTs found that the mean operation time was 158 min, with an adverse event rate of 3.5%. The R0 resection rate was 100%, and the recurrence rate was low at 0.22% (1/455) [74].

Additionally, cumulative meta‐analysis showed that ER had shorter operation times, but it was associated with a higher complication rate. However, ER showed comparable outcomes to LECS in terms of blood loss, hospital stay, costs, and recurrence rates [75]. Similarly, a network meta‐analysis revealed that LECS had longer operation times compared to ER and LR, respectively, yet the total complications were less common in LECS than in laparoscopic and ER. In contrast, no significant differences were detected in recurrence rates between LECS, LR, and ER [76]. Another meta‐analysis reported no significant differences in terms of hospital stay, operation time, adverse events, R0 resection, and conversion rate between LECS and LR. A similar outcome was also detected between LECS and ER [74]. These results indicate that LECS harnesses the individual advantages of LR and ER, reducing postoperative complications without adverse effects on oncological outcomes. The comparison of LR, ER, and LECS in the treatment of gastric GISTs is presented in Table 1.

**Table 1:** Comparison of LR, ER, and LECS surgery in the treatment of gastric GISTs.

	LR	ER	LECS
Indications	● GISTs between 2 and 5 cm● Favorable location	● Small GISTs (≤ 2 cm)● Superficial/submucosal tumors● Patients prioritizing minimal invasiveness	● Complex tumors (e.g., EGJ and posterior wall)● High‐risk ER/LR cases
Technical challenges	● En bloc resection with intact tumor pseudocapsule	● Minimally invasive mucosal/submucosal resection	● Combined laparoscopic and endoscopic guidance● Real‐time tumor localization● Reduced peritoneal contamination
Perioperative complications	● Bleeding● Anastomotic leakage● Intra‐abdominal infection● Lower rate of complications when compared to OS	● Perforation● Delayed bleeding● Gas‐related complications (pneumothorax, mediastinal emphysema)● The incidence rate of the postoperative complications of patients who underwent ER was significantly lower than that of patients who underwent LR (R, 0.4; 95% CI, 0.2–0.7) [69]	● Complication rate between LR and ER
Oncological safety	● Equal to OS in terms of long‐term oncological outcomes	● The tumor recurrence rates after ER and LR did not differ significantly	● A 100% R0 resection rate with a recurrence rate of 0.2% (1/455) was reported [74]
Postoperative outcomes	● Less operation time, less bleeding, earlier recovery, shorter hospital stays when compared to OS● The length of hospital stay for LR was 8.9 days [69]● The postoperative dietary recovery times were 3.7 days [69]	● The length of hospital stay for ER was 6.9 days [69]● The postoperative dietary recovery times were 2.9 days [69]	● The operation times are much longer in LECS than in LR and ER● The total complications were less common in LECS
Learning curve	● Requires proficiency in laparoscopic anatomy and suturing	● Demands advanced endoscopic skills (e.g., submucosal dissection)	● Needs dual expertise in laparoscopy and endoscopy
Cost‐effectiveness	● Inconclusive	● Lower initial cost but higher overall cost if complications arise	● Higher cost (dual‐modality equipment)

Abbreviations: ER, endoscopic resection; GIST, gastrointestinal stromal tumors; LECS, laparoscopic endoscopic cooperative surgery; LR, laparoscopic resection; OS, open surgery.

In our experience, the involvement of the surgical team in LECS offers several advantages for the endoscopic procedure: (i) Ultrasonic coagulating can be utilized for preligation of neurovascular around the gastric GIST; thus, the intraoperative uncontrollable bleeding can be avoided; (ii) reducing the perforation or bleeding rate by repairing the perforation or reinforcing the anastomosis site laparoscopically; (iii) local irrigation after surgery can reduce the risk of surgical site infections; (iv) shortening the operation time of EFTR by closing the incision with a stapler or suture; and (v) decreasing the incidence of gas‐related complications of ER, for excess carbon dioxide can be expelled in time during the operation. It is very important for patients with multimorbidity who are not suitable for a prolonged procedure.

For the surgical team, the endoscopic team can offer several key advantages: (i) ensuring precise incisal margin control without overresection, (ii) postoperatively re‐examine to avoid stricture and bleeding, (iii) excluding the perforation by performing an air leak test, and (iv) providing a visual location of the lesion endoscopically, especially when locating an endophytic GIST.

Although LECS has shown promising outcomes, it presents a significant learning curve due to the need for dual expertise in both laparoscopic and endoscopic techniques. The LECS procedure requires close collaboration between surgeons and endoscopists. Surgeons must be familiar with ER techniques, while endoscopists must also understand the surgical procedures involved. The integration of these modalities necessitates specialized equipment (e.g., advanced coagulation devices, staplers, and endoscopes), which may limit accessibility in certain settings. Furthermore, while LECS involves dual‐modality equipment, it may incur higher costs compared to ER and LR, particularly when complications arise (Table 1). However, the cost‐effectiveness of LECS, like that of LR, remains inconclusive and requires further investigation to fully assess its economic impact.

As a novel technique, the LECS has great potential in dealing with gastric GISTs. However, it should be noted that all available data are based on retrospective studies. Prospective studies, even randomized clinical trials with large sample sizes, are needed in the future.

## 3. Conclusions

In summary, significant advancements have challenged traditional paradigms in the surgical management of gastric GISTs. The oncological feasibility of R1 resection in selected cases offers a functional preservation strategy for tumors in unfavorable locations, while LR has proven safe and effective even for larger tumors. ER and LECS continue to evolve, providing minimally invasive options with promising outcomes, though the critical importance of en bloc resection and tumor rupture prevention during these procedures requires further validation. On the other hand, several key directions for future research deserve emphasis. There is a pressing need for prospective randomized controlled trials (RCTs) to objectively compare the long‐term efficacy of LR, ER, and LECS across different risk groups and tumor locations. Furthermore, the integration of AI in preoperative assessment holds breakthrough potential for refining risk stratification, predicting tumor behavior, and optimizing surgical planning. Finally, more studies are essential to establish standardized principles for combining different ER techniques with LECS based on tumor characteristics, with the goal of ensuring en bloc resection while maximizing minimal invasiveness. Addressing these challenges through rigorous research will be crucial for advancing the field and improving patient care.

In conclusion, while significant advances have been made in the surgical management of gastric GISTs, these developments present new challenges that demand further investigative focus.

**Nomenclature**
AI: artificial intelligence
CT: computed tomography
EFTR: endoscopic full‐thickness resection
EGJ: esophagogastric junction
EISMR: endoscopic inversion and strangulation of the muscle layer and resection
EMSLD: endoscopic mucosa‐sparing‐lateral dissection
ER: endoscopic resection
ESD: endoscopic submucosal dissection
FTRD: full‐thickness resection device
GIST: gastrointestinal stromal tumor
ICCs: interstitial cells of Cajal
LECS: laparoscopic endoscopic cooperative surgery
LIS: laparoscopic intragastric surgery
LR: laparoscopic resection
PDGFRA: platelet‐derived growth factor receptor alpha
SMCs: smooth muscle cells
STER: submucosal tunnel endoscopic resection
TKIs: tyrosine kinase inhibitors

## Author Contributions

S.Z. conceived and designed the study. S.Z. and W.S. reviewed the literature and wrote the manuscript. S.Z. substantively revised the manuscript.

## Funding

This work was funded by the Natural Science Foundation of Chongqing, China (CSTB2024NSCQ‐MSX0190), the Project of Young and Middle‐aged High‐level Medical Professional of Chongqing (YXGD202455), and the Project of Science and Technology Planning of Jiulongpo District (2023‐02‐036‐Z).

## Disclosure

All authors read and approved the manuscript. The funders had no role in the design, data collection, data analysis, and reporting of this study.

## Conflicts of Interest

The authors declare no conflicts of interest.

## Data Availability

Data sharing is not applicable to this article as no datasets were generated or analyzed during the current study.
